# The Impact of the COVID-19 Pandemic on Antimicrobial Resistance Trends in a Tertiary Care Teaching Hospital: A Ten-Year Surveillance Study

**DOI:** 10.3390/antibiotics14121179

**Published:** 2025-11-21

**Authors:** Vedrana Barišić, Tijana Kovačević, Maja Travar, Ana Golić Jelić, Pedja Kovačević, Katarina Vučićević, Dragana Milaković, Ranko Škrbić

**Affiliations:** 1 Clinical Pharmacy Department, University Clinical Centre of the Republic of Srpska, 78000 Banja Luka, Bosnia and Herzegovina or tijana.kovacevic@kc-bl.com (T.K.); dragana.milakovic@kc-bl.com (D.M.); 2 Faculty of Medicine, University of Banja Luka, 78000 Banja Luka, Bosnia and Herzegovina or maja.travar@kc-bl.com (M.T.); ana.golic@med.unibl.org (A.G.J.); pedja.kovacevic@med.unibl.org (P.K.); ranko.skrbic@med.unibl.org (R.Š.); 3 Department of Clinical Microbiology, University Clinical Centre of the Republic of Srpska, 78000 Banja Luka, Bosnia and Herzegovina; 4 Medical Intensive Care Unit, University Clinical Centre of the Republic of Srpska, 78000 Banja Luka, Bosnia and Herzegovina; 5 Department of Pharmacokinetics and Clinical Pharmacy, Faculty of Pharmacy, University of Belgrade, 11000 Belgrade, Serbia; katarina.vucicevic@pharmacy.bg.ac.rs; 6 Academy of Sciences and Arts of the Republic of Srpska, 78000 Banja Luka, Bosnia and Herzegovina; 7 Department of Pathologic Physiology, I.M. Sechenov First Moscow State Medical University, Moscow 119435, Russia

**Keywords:** drug resistance, bacterial, COVID-19, Gram-negative bacteria, anti-bacterial agents, resource-limited settings

## Abstract

**Background/Objectives:** The COVID-19 pandemic accelerated the inappropriate use of antibiotics, amplifying the global threat of antimicrobial resistance (AMR), particularly in resource-limited healthcare settings. This study investigated AMR patterns in a tertiary care hospital, focusing on the impact of the COVID-19 pandemic on invasive bacterial pathogens. **Methods:** This retrospective observational study was conducted at the University Clinical Centre of the Republic of Srpska, analyzing AMR data from invasive bacterial isolates collected between 2015 and 2024, and assessing correlations between antibiotic utilization and resistance patterns during the study periods. **Results:** Among 4718 invasive bacterial isolates, *Acinetobacter* spp. (26.7%) and *K. pneumoniae* (20.8%) were the most prevalent. A significant increase in invasive isolates was observed during the COVID-19 period, particularly for *K. pneumoniae* (*p* = 0.003), *P. aeruginosa* (*p* = 0.017), *Acinetobacter* spp. (*p* = 0.013), and *E. faecium* (*p* = 0.028). The highest multidrug-resistant (MDR) rates were observed in *Acinetobacter* spp. (97% during COVID-19) and *K. pneumoniae* (>80% post-COVID-19). Resistance increased significantly in *K. pneumoniae* to cephalosporins, fluoroquinolones, and carbapenems, and in *P. aeruginosa* and *Acinetobacter* spp. to carbapenems, while *P. aeruginosa* resistance to aminoglycosides declined. Strong correlations were found between carbapenems use and *Acinetobacter* spp. resistance (r = 0.861, *p* = 0.001), and vancomycin use and *E. faecalis* resistance (r = 0.798, *p* = 0.006). Moderate correlations were also observed between carbapenems use and resistance of *K. pneumoniae* and *P. aeruginosa*. **Conclusions:** These findings highlight the profound impact of the COVID-19 pandemic on AMR dynamics, particularly among Gram-negative pathogens, and underscore the urgent need for strengthened antimicrobial stewardship and targeted surveillance to curb the spread of MDR pathogens, especially in resource-limited hospitals.

## 1. Introduction

Despite the World Health Organization (WHO) issuing clear guidelines during the coronavirus disease (COVID-19) pandemic that recommended antibiotic use only in hospitalized patients with confirmed bacterial infections, antibiotics were often overprescribed, especially among patients with mild to moderate COVID-19 symptoms and without clinical or microbiological signs of bacterial co-infection [1]. This widespread misuse of antibiotics has contributed to the global acceleration of antimicrobial resistance (AMR), which remains one of the most urgent public health threats. Multidrug-resistant (MDR) bacteria causing life-threatening infections, particularly bloodstream infections, are becoming more common and severely limit treatment options [2,3]. These pathogens can spread within healthcare facilities and into the community, increasing the burden on healthcare systems and public health infrastructure [4]. The lack of effective antimicrobial therapies leads to longer hospital stays, higher mortality rates, and significantly increased healthcare costs [5].

Although bacterial co-infection was microbiologically confirmed in fewer than 9% of hospitalized COVID-19 patients, over 70% of patients received antibiotics, mainly due to doctors’ concerns about possible bacterial co-infection [6]. Some hospitals reported the use of more than three antibiotics for these patients [7]. However, studies have consistently shown that in most COVID-19 patients, antibiotic use offers no significant clinical benefit [8]. Human antibiotic consumption remains the main driver of AMR [9], and growing evidence suggests that COVID-19 patients with bacterial co-infections often carry highly resistant pathogens, posing a long-term risk for the spread of AMR [10]. A systematic review from 2022 estimated bacterial AMR caused 0.9 to 1.7 million deaths in 2019, ranking it among the leading causes of death worldwide [11].

AMR patterns in intensive care units (ICUs) are especially concerning, primarily because of the high rates of antibiotic use among critically ill COVID-19 patients. Those requiring invasive mechanical ventilation are particularly vulnerable to infections caused by MDR organisms, highlighting the importance of timely and accurate diagnosis of bacterial co-infections [12]. One report indicated that 54% of ICU patients developed at least one healthcare-associated infection (HAI) during their stay [13], with these superinfections being strongly linked to increased mortality [14]. Furthermore, studies have shown that about 70% of critically ill patients received at least one antibiotic, while more than half had suspected or microbiologically confirmed bacterial infections [15].

In low-resource settings (LRS), such as ours, the pandemic revealed existing vulnerabilities, including limited access to new antimicrobials, overuse, and misuse of existing resources, inadequate microbiology laboratory capacity, reliance on empirical antibiotic therapy, poor infection prevention and control measures, and subpar infrastructure for isolation and containment [16,17]. These issues, combined with a high burden of HAIs—particularly from Gram-negative pathogens—make LRS more susceptible to COVID-19-related AMR escalation [18]. Worryingly, projections estimate that by 2050, AMR could cause up to 10 million deaths annually, with 90% of these in low- and middle-income countries (LMICs) [17,19]. In the European Union, AMR already accounts for over 35,000 deaths each year [20]. Despite the vital role of AMR surveillance in infection prevention and control [21], such data are scant in LMICs due to a lack of systematic monitoring and reporting systems [22].

Given the global threat of AMR and its exacerbation during the COVID-19 pandemic, especially in vulnerable settings, ongoing surveillance and local data collection are crucial to guide stewardship efforts. In response, we carried out a study to provide essential data on local AMR trends, thereby contributing to the formulation and implementation of targeted antimicrobial stewardship strategies.

This study aimed to analyze ten-year AMR trends within a tertiary care teaching hospital, focusing on how the COVID-19 pandemic affected resistance patterns among key bacterial pathogens.

## 2. Results

Over the ten-year period, a total of 4718 invasive bacterial isolates were tested at the University Clinical Centre of the Republic of Srpska (UCC RS), with the highest annual number recorded in 2021. Most samples originated from blood cultures (97.2%), while cerebrospinal fluid accounted for only 2.8%. Among the eight monitored pathogens, *Acinetobacter* spp. and *K. pneumoniae* were the most frequently isolated, followed by *E. coli* and *S. aureus*. Nearly half of all isolates (43.0%) were obtained from ICU patients, with *Acinetobacter* spp. accounting for the largest share. Characteristics of invasive bacterial isolates and patients are summarized in Table 1.

The mean number of invasive isolates per 1000 bed-days was calculated for each of the three study periods (Table 2). Compared with the pre-COVID-19 period, there was a significant increase in the mean number of *K. pneumoniae* isolates during both the COVID-19 and post-COVID-19 periods. Conversely, the mean number of *P. aeruginosa*, *Acinetobacter* spp., and *E. faecium* isolates peaked during the COVID-19 period, showing a significant increase compared to the other periods. For *E. coli*, *S. pneumoniae*, and *E. faecalis*, no statistically significant differences were detected across the study periods. Additionally, the mean number of ICU isolates rose significantly during the COVID-19 period compared with the pre-COVID-19 period, then slightly declined in the post-COVID-19 period. Similarly, the total number of isolates increased markedly during the COVID-19 period compared to the pre-COVID-19 period and remained high in the post-COVID-19 period.

**Figure 1:**
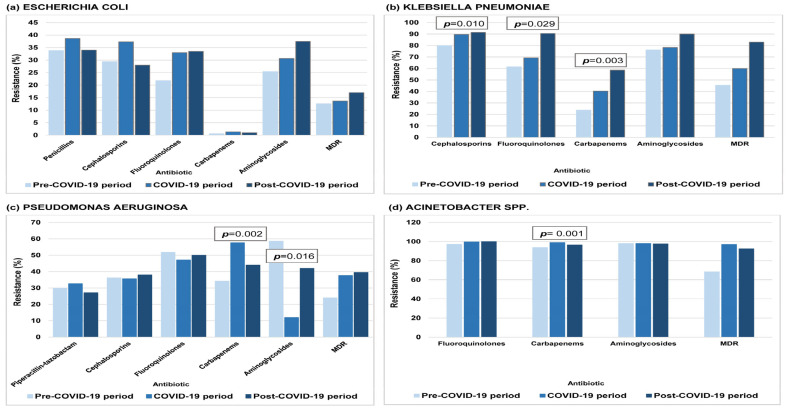
Antimicrobial resistance and proportion of MDR Gram-negative bacteria at the University Clinical Centre of the Republic of Srpska, 2015–2024 (2015–2019: pre-COVID-19; 2020–2022: COVID-19; 2023–2024: post-COVID-19). (**a**) *E. coli*. (**b**) *K. pneumoniae*. (**c**) *P. aeruginosa*. (**d**) *Acinetobacter* spp. Statistical significance was assessed using one-way ANOVA.

**Figure 2:**
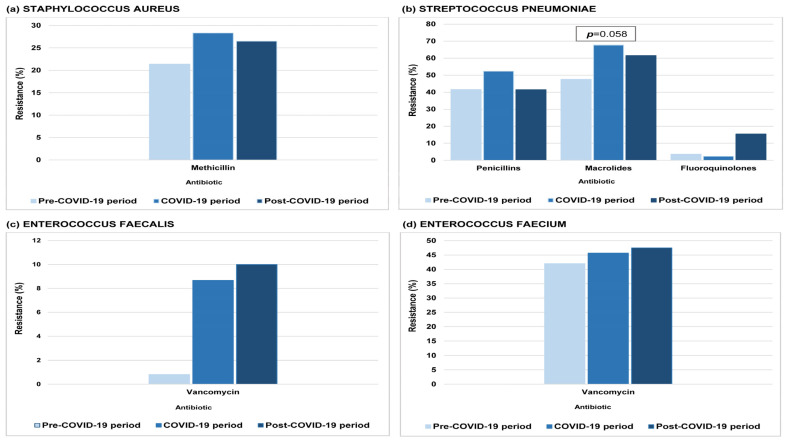
Antimicrobial resistance in Gram-positive bacteria at the University Clinical Centre of the Republic of Srpska, 2015–2024 (2015–2019: pre-COVID-19; 2020–2022: COVID-19; 2023–2024: post-COVID-19). (**a**) *S. aureus*. (**b**) *S. pneumoniae*. (**c**) *E. faecalis*. (**d**) *E. faecium.* Statistical significance was assessed using one-way ANOVA.

Figure 1 and Figure 2 depict the prevalence of AMR across the study periods for all tested pathogens. Resistance of *K. pneumoniae* to cephalosporins, fluoroquinolones, and carbapenems increased significantly during both the COVID-19 and the post-COVID-19 periods compared to pre-pandemic levels. Resistance of *P. aeruginosa* and *Acinetobacter* spp. to carbapenems also rose significantly relative to pre-pandemic levels, whereas *P. aeruginosa* resistance to aminoglycosides significantly decreased during the COVID-19 period. Resistance of *S. pneumoniae* to macrolides showed a nearly significant rise during the COVID-19 period compared to the pre-COVID-19 levels. For *E. coli*, *E. faecalis*, *E. faecium*, and *S. aureus*, no statistically significant changes in resistance levels were detected across the study periods.

The highest proportion of MDR isolates was seen in *Acinetobacter* spp., reaching 97% during the COVID-19 period, followed by *K. pneumoniae*, which exceeded 80% in the post-COVID-19 period. A moderate MDR rate was recorded in *P. aeruginosa*, while *E. coli* showed the lowest levels throughout. Although MDR rates generally increased for all Gram-negative bacteria, none of the changes were statistically significant (Figure 1).

**Figure 3:**
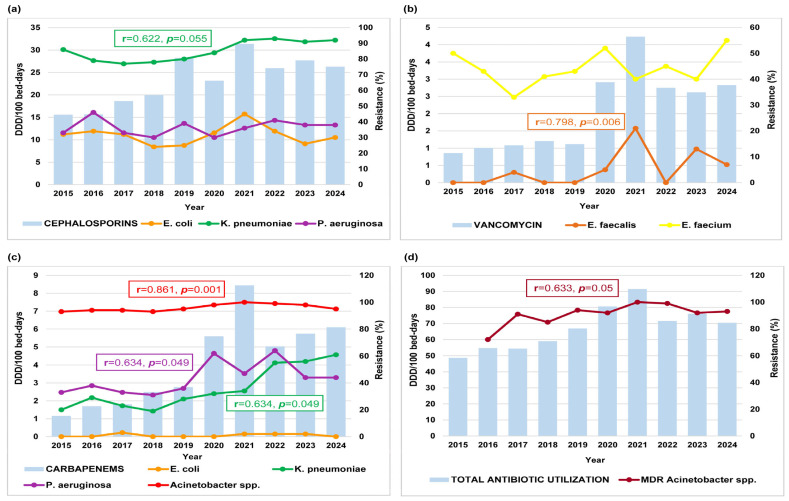
Correlations between antibiotic utilization and bacterial resistance rate at the University Clinical Centre of the Republic of Srpska, 2015–2024 (2015–2019: pre-COVID-19; 2020–2022: COVID-19; 2023–2024: post-COVID-19). (**a**) Cephalosporin use vs. resistance of *E. coli*, *K. pneumoniae*, and *P. aeruginosa*. (**b**) Carbapenem use vs. resistance of *E. coli*, *K. pneumoniae*, *P. aeruginosa*, and *Acinetobacter* spp. (**c**) Vancomycin use vs. resistance of *E. faecalis* and *E. faecium*. (**d**) Total antibiotic utilization vs. proportion of MDR *Acinetobacter* spp. isolates (missing MDR value for 2015). Values indicate Pearson’s correlation coefficients (r) with statistical significance (*p*).

Figure 3 presents only statistically significant correlations between antibiotic utilization and bacterial resistance over the ten years. A strong positive correlation was found between carbapenem use and *Acinetobacter* spp. resistance (r = 0.861; *p* = 0.001) (Figure 3c), and between vancomycin use and *E. faecalis* resistance (r = 0.798; *p* = 0.006) (Figure 3b). Moderate correlations included cephalosporin use with *K. pneumoniae* resistance (r = 0.622; *p* = 0.055) (Figure 3a), and both carbapenem use with *K. pneumoniae* (r = 0.634; *p* = 0.049), and *P. aeruginosa* resistance (r = 0.634; *p* = 0.049) (Figure 3c). Other moderate correlations, such as penicillin use with *P. aeruginosa* resistance (r = 0.466; *p* = 0.175) and macrolide use with *S. pneumoniae* resistance (r = 0.594; *p* = 0.070) did not reach statistical significance. No additional significant correlations were observed.

Correlation analysis between total antibiotic utilization and the proportion of MDR isolates showed the strongest link for *Acinetobacter* spp. (r = 0.633, *p* = 0.050) (Figure 3d), which was statistically significant. Moderate but nonsignificant positive correlations were also found for *K. pneumoniae* and *P. aeruginosa*.

## 3. Discussion

This study demonstrates that the COVID-19 pandemic was associated with a marked increase in AMR, especially for *Acinetobacter* spp. and *K. pneumoniae*, highlighting the significant impact of antibiotic use and hospital dynamics on the prevalence of MDR pathogens. The overall number of invasive bacterial isolates increased substantially during the pandemic, and the trend persisted in the post-pandemic years. The increase was particularly notable for *K. pneumoniae*, while *P. aeruginosa*, *Acinetobacter* spp., and *E. faecium* peaked during the pandemic. These findings highlight the disproportionate burden of Gram-negative bacteria in critically ill patients and are consistent with reports from other tertiary care hospitals [12,23,24]. Similar patterns of invasive isolates have been reported in neighboring countries such as the Republic of Serbia, the Republic of Croatia, and Hungary [25,26].

The number of isolates from ICU patients increased markedly during the pandemic, underscoring the role of intensive care settings in driving AMR trends. Since a large share of isolates came from ICU patients, this unit appears to be one of the key drivers in hospital-wide resistance patterns. However, AMR in the ICU cannot be solely attributed to ICU practices, as many patients were exposed to antibiotics before admission from other wards or primary care settings. Additionally, the high volume of microbiological samples collected from ICU patients contributes to the higher detection of resistant organisms in these units.

During the study period, *K. pneumoniae* showed a clear upward trend in resistance to cephalosporins, fluoroquinolones, and carbapenems, especially during the COVID-19 and post-COVID-19 periods. This aligns with European and global data identifying *K. pneumoniae* as one of the major drivers of hospital-acquired infections [27,28]. Similar patterns have been reported in low-resource settings, such as Nigeria and South Africa, where resistance is rising and expected to increase further by 2026. A meta-analysis of 19 studies also reported high resistance rates of *K. pneumoniae* to cephalosporins (>50%), rising resistance to carbapenems (~35%), and moderate to high resistance to fluoroquinolones (2–45.6%) [29].

Resistance of *Acinetobacter* spp. to carbapenems rose markedly during the pandemic, mirroring global observations that identify this pathogen as a particularly serious threat in ICUs. Other studies have linked this trend to invasive procedures and prolonged broad-spectrum antibiotic therapy in critically ill patients [30,31]. A nearby hospital reported 66% resistance of *A. baumannii* to meropenem in 2021 [32]. Surveillance data from Serbia and the Western Balkans have also pointed to high and increasing carbapenem resistance in *Acinetobacter* spp., along with ongoing gaps in AMR monitoring [33,34].

The resistance of *P. aeruginosa* to carbapenems increased during the pandemic, while resistance to aminoglycosides declined. Conversely, international studies have often found decreasing resistance rates in *P. aeruginosa* during the pandemic, including for carbapenems and aminoglycosides [35,36,37]. These discrepancies likely stem from differences in prescribing patterns and the pathogen’s resistance mechanisms.

The resistance of *S. pneumoniae* to macrolides increased significantly during the pandemic, consistent with reports of macrolide overuse for respiratory infections in that period, raising concerns about the potential loss of macrolide efficacy in treating pneumococcal disease [38,39,40]. Nevertheless, regional differences have been documented, with declining macrolide resistance reported in the Republic of Slovenia [41] and stable susceptibility levels observed in Japan [42]. Regarding enterococci, vancomycin resistance in both species displayed a rising tendency without reaching statistical significance, which is consistent with previously published data [43,44,45,46].

In contrast, no significant changes in resistance were observed for *E. coli* and *S. aureus*. These findings mirror reports from other studies, which documented only slight increases in *E. coli* resistance and largely unchanged resistance in *S. aureus* [27,47].

All Gram-negative bacteria in this study exhibited rising MDR trends, with the highest levels observed in *Acinetobacter* spp. and *K. pneumoniae*, both deemed critical-priority pathogens by WHO due to their significant global health threat [48]. This matches Slovenian data indicating increased MDR in bloodstream isolates during the pandemic, though without statistical significance [49]. Similarly, reports from LMCIs, including Egypt and Southeast Asia, noted increases in MDR *K. pneumoniae*, albeit to a lesser extent than in our setting [50,51]. A Brazilian study identified carbapenem-resistant *K. pneumoniae* as the leading MDR pathogen after the pandemic [52].

The correlation analyses further underscored the role of antibiotic pressure in shaping AMR patterns. Notably, a strong association was found between carbapenem use and *Acinetobacter* spp. resistance, suggesting its possible role as a driver of carbapenem resistance in hospitals [53,54]. Likewise, cephalosporin and carbapenem consumption were associated with increasing resistance in *K. pneumoniae*, which is consistent with global data that recognize this pathogen as a major reservoir and disseminator of resistance determinants [54,55,56]. For *P. aeruginosa*, our findings illustrate its highly variable responses to antimicrobial pressure, aligning with reports that demonstrated the association between carbapenem exposure and increased resistance rates [57] and confirming that carbapenem-resistant *P. aeruginosa* remains widespread worldwide [58]. Our recent study demonstrated a significant rise in antibiotic utilization during the COVID-19 period, especially of carbapenems and cephalosporins, which remained elevated in the post-pandemic years [59]. This upward trend in antibiotic use, consistent with global reports of increased empirical prescribing during the pandemic [6,60], likely contributed to the parallel rise in resistance to these antimicrobial classes, particularly among *K. pneumoniae* and *Acinetobacter* spp., observed in the present study. A moderate yet non-significant correlation was also observed between penicillin use and *P. aeruginosa* resistance, supporting findings from comparative studies showing increased resistance with the use of the antipseudomonal agent piperacillin-tazobactam [61].

Although resistance of *E. faecalis* to vancomycin did not reach statistical significance across the study periods, vancomycin consumption was clearly associated with increased resistance, particularly during 2021. The near-significant association between macrolide use and *S. pneumoniae* resistance fits with reports of increased macrolide prescribing for respiratory tract infections, raising global concerns about diminished effectiveness [42,43,44]. Collectively, these findings highlight antimicrobial pressure as a key driver of resistance evolution during the pandemic, especially in developing countries [62]. When total antibiotic utilization was analyzed, the strongest association was observed with MDR *Acinetobacter* spp., followed by moderate associations with MDR *K. pneumoniae* and MDR *P. aeruginosa*.

This study has several limitations that should be acknowledged. First, the single-center scope limits broad applicability, as antimicrobial resistance dynamics may differ across hospitals and healthcare systems. Second, the retrospective design prevents addressing patient-level factors, like previous antibiotic exposure, comorbidities, or length of hospital stay, which may have influenced the development of resistance. Third, antibiotic utilization was measured at the hospital level, without stratification by department or indication, limiting insights into prescribing practices in specific clinical contexts. Fourth, duplicate isolates from the same patient were not excluded, which may have led to a slight overrepresentation of certain resistance patterns. Fifth, although correlations between antibiotic use and resistance were explored, causality cannot be inferred due to the study design, which assesses population-level rather than individual-level associations. Finally, microbiological data were restricted to invasive isolates, which may not fully represent resistance trends in non-invasive infections.

Despite these limitations, the ten-year timeframe, inclusion of multiple invasive pathogens, and correlation with antibiotic use provide valuable insights into the dynamics of antimicrobial resistance in a resource-limited tertiary care setting. Moreover, this study offers a comprehensive overview of AMR trends and antibiotic utilization, contributing important data to bolster antimicrobial resistance surveillance efforts in Bosnia and Herzegovina, where such data remain scarce.

## 4. Materials and Methods

### 4.1. Study Design and Setting

This retrospective observational study was conducted at the UCC RS, which is the biggest university-affiliated tertiary care teaching hospital in the Republic of Srpska, Bosnia and Herzegovina, with 1200 beds. The study analyzed annual AMR data collected over ten years, from January 2015 to December 2024.

The study protocol was approved by the Ethics Committee of the UCC RS (approval no. 01-314 19-120-2/25) and the Ethics Committee for human and biological material research of the Faculty of Medicine, University of Banja Luka (approval no. 18/4. 67/25).

### 4.2. Data Collection

To assess the impact of the COVID-19 pandemic on AMR, data were grouped into three time periods: pre-COVID-19 (from 2015 to 2019), COVID-19 (from 2020 to 2022), and post-COVID-19 (from 2023 to 2024).

The study focused on invasive bacterial isolates (blood and cerebrospinal fluid) from hospitalized patients routinely tested at the Department of Clinical Microbiology of UCC RS during the study period. The isolates were identified using automated blood culture systems, BACT/ALERT^®^ VIRTUO^®^ (bioMérieux S.A., Marcy-I’Ėtoile, France) and BD BACTEC^TM^ FX (Becton, Dickinson and Company, New Jersey, USA). Antimicrobial susceptibility testing (AST) fulfilled European Committee on Antimicrobial Susceptibility Testing (EUCAST) guidelines [63], with clinical breakpoints relevant to each year. Resistance phenotypes were classified in accordance with the Central Asian and European Surveillance of Antimicrobial Resistance (CAESAR) [4] and the European Antimicrobial Resistance Surveillance Network (EARS-Net) [26] protocols. Isolates were classified as resistant when non-susceptible (I or R) according to EUCAST definitions [63].

The analysis included eight key bacterial pathogens: *Escherichia coli* (*E. coli*), *Klebsiella pneumoniae* (*K. pneumoniae*), *Pseudomonas aeruginosa* (*P. aeruginosa*), *Acinetobacter* spp. (*Acinetobacter*), *Staphylococcus aureus* (*S. aureus*), *Streptococcus pneumoniae* (*S. pneumoniae*), *Enterococcus faecalis* (*E. faecalis*), and *Enterococcus faecium* (*E. faecium*).

For each pathogen, the total number of tested invasive isolates and the percentage of resistance were recorded annually, with consistent antibiotic panels throughout the study to ensure data comparability across years. Duplicate isolates from the same patient were not excluded. Resistance was assessed for the following antibiotic classes and agents:

- *E. coli*: penicillins (ampicillin, amoxicillin, amoxicillin-clavulanic acid, piperacillin-tazobactam), third-generation cephalosporins (cefotaxime, ceftriaxone), fluoroquinolones (ciprofloxacin, levofloxacin, ofloxacin), carbapenems (meropenem, imipenem), and aminoglycosides (gentamicin, tobramycin);
- *K. pneumoniae*: third-generation cephalosporins (cefotaxime, ceftriaxone), fluoroquinolones (ciprofloxacin, levofloxacin, ofloxacin), carbapenems (meropenem, imipenem), and aminoglycosides (gentamicin, tobramycin);
- *P. aeruginosa*: third-generation cephalosporin (ceftazidime), fluoroquinolones (ciprofloxacin, levofloxacin), carbapenems (meropenem, imipenem), aminoglycosides (gentamicin, amikacin, tobramycin), and piperacillin-tazobactam;
- *Acinetobacter* spp.: fluoroquinolones (ciprofloxacin, levofloxacin, ofloxacin), carbapenems (meropenem, imipenem), and aminoglycosides (gentamicin, tobramycin);
- *S. aureus*: methicillin (MRSA);
- *S. pneumoniae*: resistance to penicillin, fluoroquinolones (levofloxacin, moxifloxacin), and macrolides (erythromycin, clarithromycin, azithromycin);
- *E. faecalis* and *E. faecium*: resistance to vancomycin.

Resistance levels were evaluated for each antibiotic or class, in line with CAESAR surveillance protocols. The proportion of MDR isolates was calculated only for Gram-negative bacteria, with MDR defined as resistance to at least one agent in three or more antimicrobial classes [4].

To estimate AMR at the antibiotic class level, a weighted average of resistance rates for individual antibiotics within each group was calculated. This method accounts for the varying number of isolates tested per antibiotic, thus preventing bias in favor of more or less frequently tested agents. Equation (1) used was:

(1) $$Group\;Resistance(\%)=\frac{\sum_{i=1}^{n}\left(Ri\times Ni\right)}{\sum_{i=1}^{n}Ni}$$

where R*i* represents the resistance rate for antibiotic *i*, N*i* is the number of isolates tested for agent *i*, and *n* is the total number of antibiotics included in the group.

This method improves the accuracy of overall resistance within an antibiotic class by emphasizing antibiotics with more tested isolates. This methodology has been documented and illustrated in surveillance studies, such as those assessing nosocomial resistance trends among Gram-negative bacteria, where authors described the “overall R%” as the weighted average of individual resistance rates, appropriately accounting for isolate counts per antibiotic [64].

Additionally, the annual isolate density rate, expressed as the number of invasive bacterial isolates per 1000 patient-days, was calculated. This approach enabled us to account for variations in hospital occupancy over time and to assess whether the rate of isolate detection increased relative to patient exposure. To further contextualize these findings, annual bed occupancy rates were also presented to reflect differences in hospital capacity utilization.

### 4.3. Antibiotic Utilization

The impact of antibiotic (ATC group J01) utilization on AMR in all three study periods was analyzed. Antibiotic utilization was expressed as defined daily doses per 100 bed-days (DDD/100 BD), as reported in a previously published study [59]. To determine the correlation between resistance levels of specific pathogens and antibiotic use, in addition to the data presented in that study, extra data on the utilization of individual antibiotics were used.

### 4.4. Statistical Analyses

The data was recorded and analyzed using Microsoft^®^ Excel^®^ Version 2013 (Microsoft Corporation, Redmond, WA, USA) and IBM^®^ SPSS^®^ Version 26.0 (IBM Corp., Armonk, NY, USA) statistical program. Descriptive statistics were used to describe rates of bacterial resistance. Continuous variables were presented as the mean with standard deviation (SD) for normally distributed data and as the median with interquartile range (IQR) for non-normally distributed data. Normality of data distribution was assessed using the Kolmogorov–Smirnov and Shapiro–Wilk tests, and homogeneity of variances was evaluated with Levene’s test. Categorical variables were reported as absolute numbers or percentages. One-way ANOVA was used to compare the continuous variables across groups. When ANOVA indicated significant differences, pairwise comparisons were performed using Tukey’s honestly significant difference (HSD) post hoc test. To explore the correlation between antibiotic consumption and AMR trends, Pearson’s correlation coefficient was calculated. The statistical significance level was set at 0.05, with a 95% confidence interval.

## 5. Conclusions

Our findings demonstrate that the COVID-19 pandemic significantly accelerated AMR progression, especially among Gram-negative bacteria like *Acinetobacter* spp. and *K. pneumoniae.* The strong associations observed between antibiotic consumption and resistance patterns highlight the central role of antimicrobial pressure in shaping resistance dynamics. These results emphasize the importance of antimicrobial stewardship, particularly in high-risk environments such as ICUs. In resource-limited hospitals, targeted surveillance and optimized prescribing practices are crucial to reducing MDR pathogen burden and maintaining antibiotic efficacy.

## Figures and Tables

**Table 1:** Baseline characteristics of patients and invasive bacterial isolates during the ten-year study period at the University Clinical Centre of the Republic of Srpska.

	*E. coli*	*K. pneumoniae*	*P. aeruginosa*	*Acinetobacter* spp.	*S. aureus*	*S. pneumoniae*	*E. faecalis*	*E. faecium*	Total
Number of isolates, n (%)	751 (15.9)	982 (20.8)	334 (7.1)	1258 (26.7)	711 (15.1)	122 (2.6)	291 (6.1)	269 (5.7)	4718 (100)
Isolate source, n									
Blood	748	971	329	1215	700	87	280	258	4588
Cerebrospinal fluid	3	11	5	43	11	35	11	11	130
Sex, n									
Male	279	655	221	816	470	79	194	172	2886
Female	470	327	113	441	241	42	97	97	1828
Unknown	2	0	0	1	0	1	0	0	4
Age (years), n									
0–4	80	137	15	91	61	14	21	22	441
5–19	4	12	7	5	10	15	3	3	59
20–64	318	417	161	515	321	55	131	100	2018
≥65	349	414	150	646	317	38	135	144	2193
Unknown	0	2	1	1	2	0	1	0	7
Hospital department, n									
Emergency	144	26	9	1	98	12	31	2	323
Hematology or oncology	34	62	33	25	31	12	10	13	220
Infectious disease	274	133	16	21	123	52	43	15	677
Internal medicine	120	133	73	46	183	9	98	57	719
Obstetrics or gynecology	13	6	0	1	6	0	8	0	34
Surgery	10	27	8	22	50	0	13	11	141
Urology	28	30	3	1	8	0	14	9	93
Intensive care unit	48	424	174	1041	139	15	50	136	2027
Pediatrics or neonatal	44	42	11	22	39	22	7	9	196
Pediatrics or neonatal intensive care unit	35	98	7	72	29	0	15	14	270
Other	1	1	0	1	5	0	1	1	10
Unknown	0	0	0	5	0	0	1	2	8

**Table 2:** Changes in the number of bacterial isolates at the University Clinical Centre of the Republic of Srpska, from 2015 to 2024 (2015–2019: pre-COVID-19; 2020–2022: COVID-19; 2023–2024: post-COVID-19).

	Number of Isolates/1000 BD, Mean (±SD)			*p*-Value ^1^
	Pre-COVID-19 Period	COVID-19 Period	Post-COVID-19 Period	
*E. coli*	0.19 (0.06)	0.16 (0.03)	0.26 (0.04)	0.178
*K. pneumoniae*	0.15 (0.05)	0.31 (0.07)	0.40 (0.01)	0.003
*P. aeruginosa*	0.06 (0.04)	0.15 (0.04)	0.08 (0.01)	0.017
*Acinetobacter* spp.	0.14 (0.08)	0.77 (0.38)	0.17 (0.04)	0.013
*S. aureus*	0.15 (0.07)	0.20 (0.02)	0.25 (0.01)	0.138
*S. pneumoniae*	0.03 (0.02)	0.03 (0.01)	0.04 (0.00)	0.598
*E. faecalis*	0.07 (0.03)	0.10 (0.03)	0.06 (0.01)	0.396
*E. faecium*	0.05 (0.03)	0.12 (0.03)	0.06 (0.01)	0.028
ICU	0.25 (0.13)	1.08 (0.49)	0.45 (0.09)	0.015
Total	0.82 (0.32)	1.77 (0.44)	1.29 (0.03)	0.016

^1^ One-way ANOVA test; BD—bed-days; ICU—intensive care unit; SD—standard deviation.

## Data Availability

The data presented in this study are available from the corresponding author upon reasonable request.
